# Supplementary material for: Resolving the full spectrum of human genome variation using Linked-Reads
Source: Genome Res. 2019 Apr;29(4):635–45. doi: 10.1101/gr.234443.118 (PMC6442396; doi:10.1101/gr.234443.118)
Supplement: Supplemental Material [file supp_gr.234443.118_Supplemental_longranger-2.2.zip › longranger-2.2.tar.bz2!/lr2.2-public/lib/stan/cmdstan/stan/doc/api/html/check__consistent__sizes_8hpp_source.html]

Stan: src/stan/math/error\_handling/check\_consistent\_sizes.hpp Source File


|  |  |
| --- | --- |
|  | Stan  2.5.0  probability, sampling & optimization |


- Main Page
- Related Pages
- Namespaces
- Classes
- Files

- File List
- File Members

All Classes Namespaces Files Functions Variables Typedefs Enumerations Enumerator Friends Macros Pages

- src
- stan
- math
- error\_handling

check\_consistent\_sizes.hpp

Go to the documentation of this file.

1 #ifndef STAN\_\_MATH\_\_ERROR\_HANDLING\_\_CHECK\_CONSISTENT\_SIZES\_HPP

2 #define STAN\_\_MATH\_\_ERROR\_HANDLING\_\_CHECK\_CONSISTENT\_SIZES\_HPP

3

4 #include <stan/math/error\_handling/check\_consistent\_size.hpp>

5 #include <stan/math/error\_handling/dom\_err.hpp>

6 #include <stan/meta/traits.hpp>

7

8 namespace stan {

9  namespace math {

10

11  // NOTE: this will not throw if nan is passed in.

12  template <typename T1, typename T2, typename T\_result>

13  inline bool check\_consistent\_sizes(const char\* function,

14  const T1& x1,

15  const T2& x2,

16  const char\* name1,

17  const char\* name2,

18  T\_result\* result) {

19  size\_t max\_size = std::max(size\_of(x1),

20  size\_of(x2));

21  return check\_consistent\_size(max\_size,function,x1,name1,result)

22  && check\_consistent\_size(max\_size,function,x2,name2,result);

23  }

24

25  template <typename T1, typename T2, typename T3, typename T\_result>

26  inline bool check\_consistent\_sizes(const char\* function,

27  const T1& x1,

28  const T2& x2,

29  const T3& x3,

30  const char\* name1,

31  const char\* name2,

32  const char\* name3,

33  T\_result\* result) {

34  size\_t max\_size = std::max(size\_of(x1),

35  std::max(size\_of(x2),size\_of(x3)));

36  return check\_consistent\_size(max\_size,function,x1,name1,result)

37  && check\_consistent\_size(max\_size,function,x2,name2,result)

38  && check\_consistent\_size(max\_size,function,x3,name3,result);

39  }

40  template <typename T1, typename T2, typename T3, typename T4, typename T\_result>

41  inline bool check\_consistent\_sizes(const char\* function,

42  const T1& x1,

43  const T2& x2,

44  const T3& x3,

45  const T4& x4,

46  const char\* name1,

47  const char\* name2,

48  const char\* name3,

49  const char\* name4,

50  T\_result\* result) {

51  size\_t max\_size = std::max(size\_of(x1),

52  std::max(size\_of(x2),

53  std::max(size\_of(x3), size\_of(x4))));

54  return check\_consistent\_size(max\_size,function,x1,name1,result)

55  && check\_consistent\_size(max\_size,function,x2,name2,result)

56  && check\_consistent\_size(max\_size,function,x3,name3,result)

57  && check\_consistent\_size(max\_size,function,x4,name4,result);

58  }

59

60  }

61 }

62 #endif

dom\_err.hpp

check\_consistent\_size.hpp

stan::math::max

double max(const double a, const double b)

**Definition:** max.hpp:7

traits.hpp

stan::math::check\_consistent\_size

bool check\_consistent\_size(size\_t max\_size, const char \*function, const T &x, const char \*name, T\_result \*result)

**Definition:** check\_consistent\_size.hpp:13

stan::size\_of

size\_t size\_of(const T &x)

**Definition:** traits.hpp:186

stan::math::check\_consistent\_sizes

bool check\_consistent\_sizes(const char \*function, const T1 &x1, const T2 &x2, const char \*name1, const char \*name2, T\_result \*result)

**Definition:** check\_consistent\_sizes.hpp:13

stan::max\_size

size\_t max\_size(const T1 &x1, const T2 &x2)

**Definition:** traits.hpp:191


---

[ Stan Home Page ]

*© 2011–2014,
Stan Development Team.*
